# New York City Mortality

**Published:** 1857-08

**Authors:** 


### NEW YORK CITY MORTALITY.

| | 1853. | 1854. | 1855. | 1856. |
|---|---|---|---|---|
| DEATHS from all causes, | 22,702 | 28,568 | 22,787 | 21,658 |
| " " Throat and Lungs, | 5,648 | 6,174 | 5,770 | 5,382 |
| " " Consumption only, | 2,739 | 3,032 | 2,624 | 2,478 |

IT was procured to be stated in a certain class of newspapers, in the early part of last year, that the number of deaths from consumption, during 1855, was twenty-five per cent., or one fourth less than during 1854, and that this diminution was owing to the efficacy of Medicated Inhalation. But on examining the figures, it will be seen that there was a difference of four hundred and eight, or about fourteen per cent. Still the statement was originated in New York, and published all over the land. But there were twenty per cent. less deaths in New York during 1855, than in the preceding year, from all causes, so that in reality, there was not as great a diminution of deaths from consumption, as there was in all diseases. This greater fatality in consumption might be attributed to Medicated Inhalation, with some show of truth.

Medicated Inhalation was in full blast, in New York, in the latter part of 1855. In comparing the consumptive mortality of the first quarters of 1854, '5 and '6, a steady diminution was observed, of ten per cent., and then of twenty-five per cent., and the statement was made at the time, that "additional skill in the application of Inhalation for lung diseases, acquired by observation and experience, would afford the most conclusive proof of its remedial power, for good." As it would require months and years to verify these statements, time was afforded to reap a rich harvest from this false gloss, and now what does time reveal?

| Consumption Deaths in N. Y., | 1854. | 1855. | 1856. | 1857. |
|---|---|---|---|---|
| For Jan., Feb., March | 843 | 776 | 580 | 779. |
| All Diseases for 1st quarter | 6,282 | 5,681 | 4,598 | 5,851. |

By which it will be seen, that the deaths from consumption during the first quarter of the present year, were greater than during that of 1856, by thirty-four per cent.; so that "additional skill in the observation and practice of Inhalation," for the cure of consumption, has not diminished the mortality of that disease, but that mortality has increased in spite of that skill, by the large percentage just named. This presents a different view of the efficacy of Medicated Inhalation, from that given in the following item, which has appeared in many papers.

"The *New York Herald* says, that Dr. ——, of that city, has been accomplishing the most extraordinary results in the treatment of consumption, by *Inhalation*, decreasing the mortality more than *one thousand*, in the past two years. Deaths from consumption, in New York, for 1854, were 3,032, for '55 were 2,624, for 1856 were 2,357, showing an actual saving of life, truly miraculous, when we consider that this disease has hitherto been regarded as hopelessly fatal."

How "more than one thousand lives" were saved, during '55 and '56, by Inhalation, is certainly not demonstrated by the above figures; and it is a striking illustration of the blind carelessness with which many papers are made up. If the number of consumptive deaths was diminished in New York, for 1856, by 267, or ten per cent., through the agency of Inhalation, how can we account for the fact, that during the first quarter of the present year, the number of deaths by consumption was one hundred and ninety-nine greater than for the corresponding quarter of last year, that is thirty-four per cent.

Reflecting men will readily conclude from these statements, that the diminution of the number of deaths by consumption, was from accidental causes, that the later increase of that kind of mortality is from accidental causes, and that *Medicated Inhalation* has no appreciable agency in the changes which these figures indicate.

If we were disposed to resort to the *ad captandum* argument, we might insist that this increased mortality, of over thirty-four per cent. of the first quarter of 1857 over 1856, was owing to the large number of persons drawn to New York by the excitement of false hopes, and have only come to die by scores and hundreds, while Inhaling. Will the newspapers which lent their aid in exciting these groundless hopes in their confiding patrons, publish this correction, and let them see how things really stand? The real truth being, that consumption with other diseases, increases or diminishes in the main, with the increase or diminution of the deaths from all causes. To claim a diminution from the agency of the practice of a single individual, exhibits a recklessness of truth, and an impudence of assumption, which richly merit the contempt of all reflecting men.

